# Early Occipitocervical Fusion Surgery in a Rare Clinical Encounter of Non-traumatic Atlantooccipital Subluxation (AOS) in Down Syndrome (Trisomy 21)

**DOI:** 10.7759/cureus.67713

**Published:** 2024-08-25

**Authors:** Lim Hong Ng, Jin Aun Tan, Suffian Sabri, Azmi Baharuddin, Mohd Hisam Muhamad Ariffin

**Affiliations:** 1 Orthopaedics and Traumatology, Universiti Kebangsaan Malaysia Medical Centre, Kuala Lumpur, MYS; 2 Orthopaedics and Traumatology, Pusat Perubatan Universiti Kebangsaan Malaysia, Kuala Lumpur, MYS

**Keywords:** atlanto-occipital c2-c3 fusion, trisomy of 21, cervical spine anomalies, cervical spine alignment, pediatric cervical spine, pediatric spine surgery, spine and pediatrics orthopedics, occipito-cervical fusion surgery, atlanto-axial subluxation, down's syndrome

## Abstract

Subluxation of the atlantooccipital joint in patients with underlying Down syndrome is an extremely rare orthopedic condition. The condition can pose life-threatening risks if not promptly diagnosed and treated in the early stage. Yet, there have been documented cases of survival following atlantooccipital subluxation or dislocation. Atlantooccipital subluxation (AOS) is usually identified during screening in children with Down syndrome for atlantoaxial subluxation (AAS). Therefore, careful evaluation of the atlantooccipital joint from radiographs is also essential. It is crucial to emphasize the clinical significance of AOS.

Here the authors present the case of a fifteen-year-old girl with underlying Down syndrome (trisomy 21) who survived a sudden onset of non-traumatic atlantooccipital subluxation with spinal cord compression. There are only a few cases were reported in patients with Down syndrome (trisomy 21) and only two cases with surgically treated atlantooccipital (C0C1) subluxation have been reported.

This case is of particular interest as it represents the first reported case of atlantooccipital (C0C1) subluxation with spinal cord compression in Down syndrome that underwent occipitocervical fusion surgery during the acute presentation, resulting in significant neurological recovery. Her neurology symptoms and physical functions showed remarkable improvement post-surgery, and she is doing well at the one-year follow-up in the clinic. Early surgery during acute presentation in this case resulted in good surgical outcomes and improved patient quality of life.

## Introduction

Spitzer et al. were the first to document atlantoaxial subluxation or instability in a patient with Down syndrome in 1961, and subsequent research has revealed its common occurrence with 14 out of 70 children affected in one series [1]. Subsequent research by Semine et al. found abnormalities of the C1-C2 articulation in 18% of 85 children with Down syndrome. Atlantooccipital dislocation is an exceptionally rare occurrence observed in living patients [2].

To date, only nine cases attributed to trauma have been documented in the literature, as reported by Powers et al. in 1979. Among these cases, six individuals managed to survive the injury [3]. Atlantooccipital dislocation due to causes other than trauma appears to be exceptionally rare, based on our limited review of the literature. Nontraumatic atlantooccipital dislocations can arise secondary to various conditions, including extensive erosive calcium pyrophosphate dihydrate deposition disease, rheumatoid arthritis, congenital skeletal abnormalities, and infections.

Only a few reports documenting this issue in individuals with Down syndrome can be found in the literature. Atlantooccipital subluxation (AOS) in association with Down syndrome was reported first by Hungerford et al. in 1981 [4]. The exact cause of the atlantooccipital subluxation in our case remains uncertain, given the absence of any history of trauma or other preceding factors. Our patient represents the first documented case of non-traumatic atlantooccipital (C0C1) subluxation with spinal cord compression in Down syndrome that underwent occipitocervical fusion surgery during the acute presentation (two weeks from the onset of neurological presentation), resulting in a favorable surgical outcome and neurological recovery.

## Case presentation

A fifteen-year-old girl with underlying Down syndrome (trisomy 21) presented to us with acute neurological symptoms that have been worsening over the past two weeks. The father noted that the patient started having bilateral upper limbs mild shivering for six months, followed by a sudden onset of bilateral upper limb weakness two weeks before admission. These symptoms have significantly affected her daily activities. There was no history of falls or trauma at home before admission. The father decided to seek treatment immediately when the patient developed two episodes of urinary incontinence and one episode of bowel incontinence, accompanied by a floppy neck posture. Although the patient is still able to ambulate unaided, she exhibits clumsiness and moves at a very slow pace.

The physical examination revealed an alert girl with Down syndrome facies, lying on the bed with cervical collar protection. The muscle power assessment (MRC Scales) of her upper and lower limbs indicated weak motor power, graded at only three to four for all muscles. The sensory examination could not be accurately elicited due to the patient's limited understanding. Deep tendon reflexes were increased bilaterally in both upper and lower limbs. Clonus was observed in the bilateral lower limbs, while Babinski's sign was negative.

**Figure 1:**
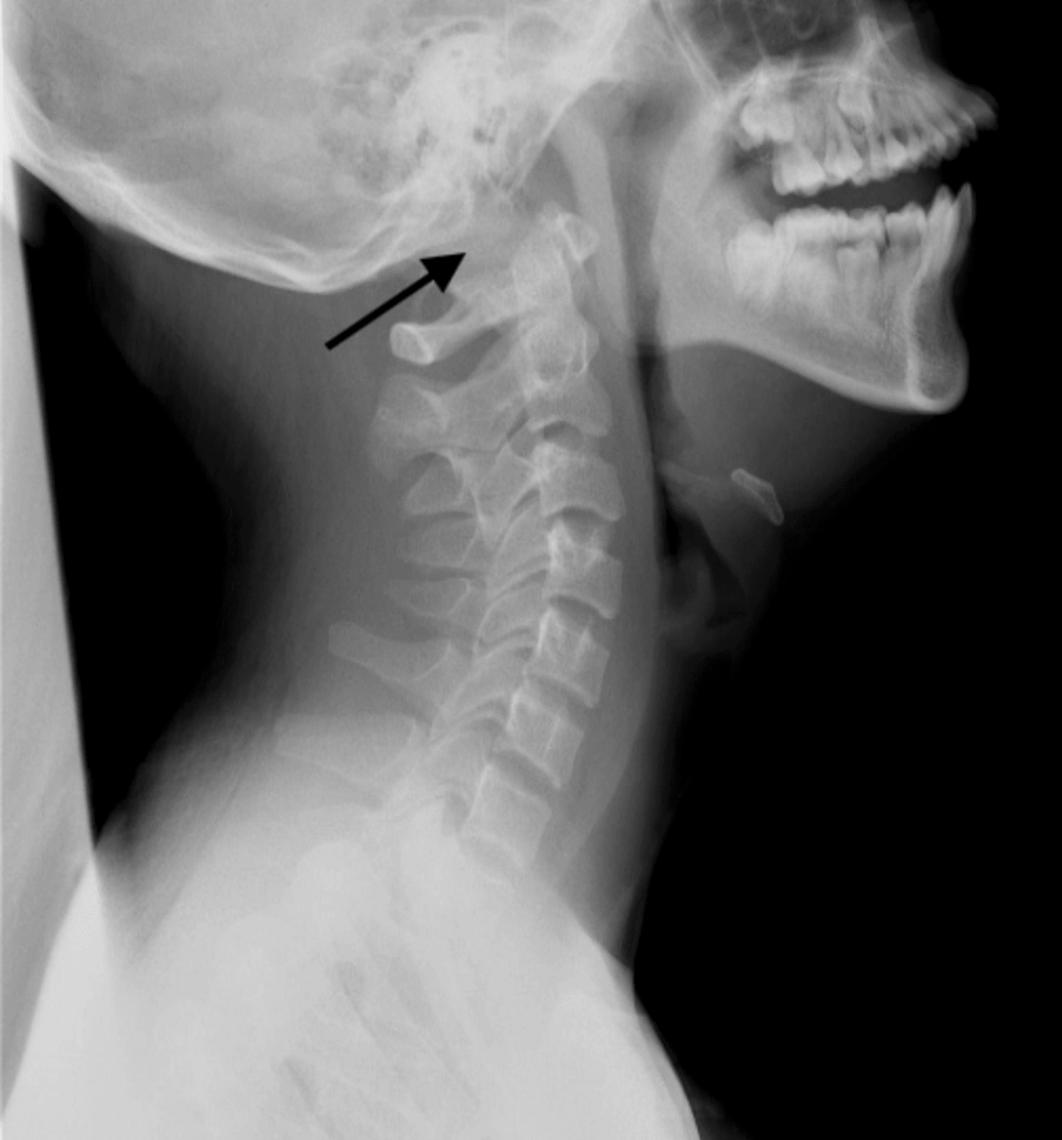
Preoperative lateral view of cervical x-ray for the patient. The x-ray showed subluxation of the atlantooccipital joint (arrow).

**Figure 2:**
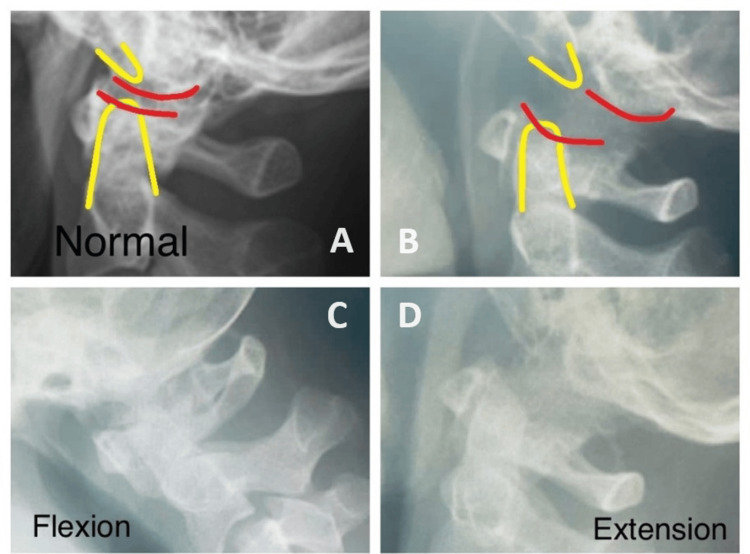
Comparison of lateral view cervical x-ray of the patient in neutral (B), flexion (C), and extension (D) with the ideally normal x-ray (A). Extension and neutral views show atlantooccipital subluxation (AOS). Flexion view shows partial reduction of AOS.

**Figure 3:**
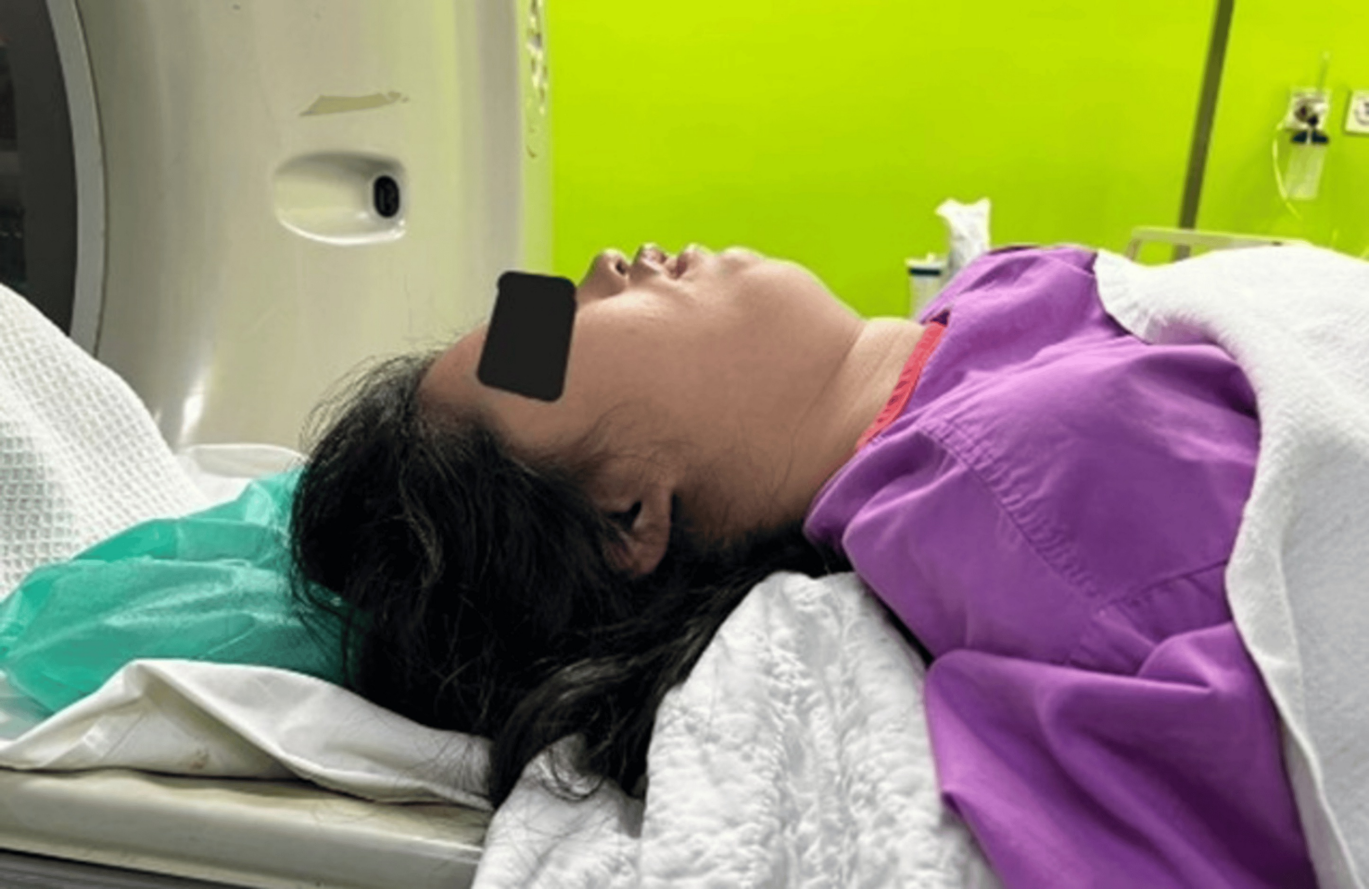
The patient was carefully transferred for imaging investigation with the floppy neck.

**Figure 4:**
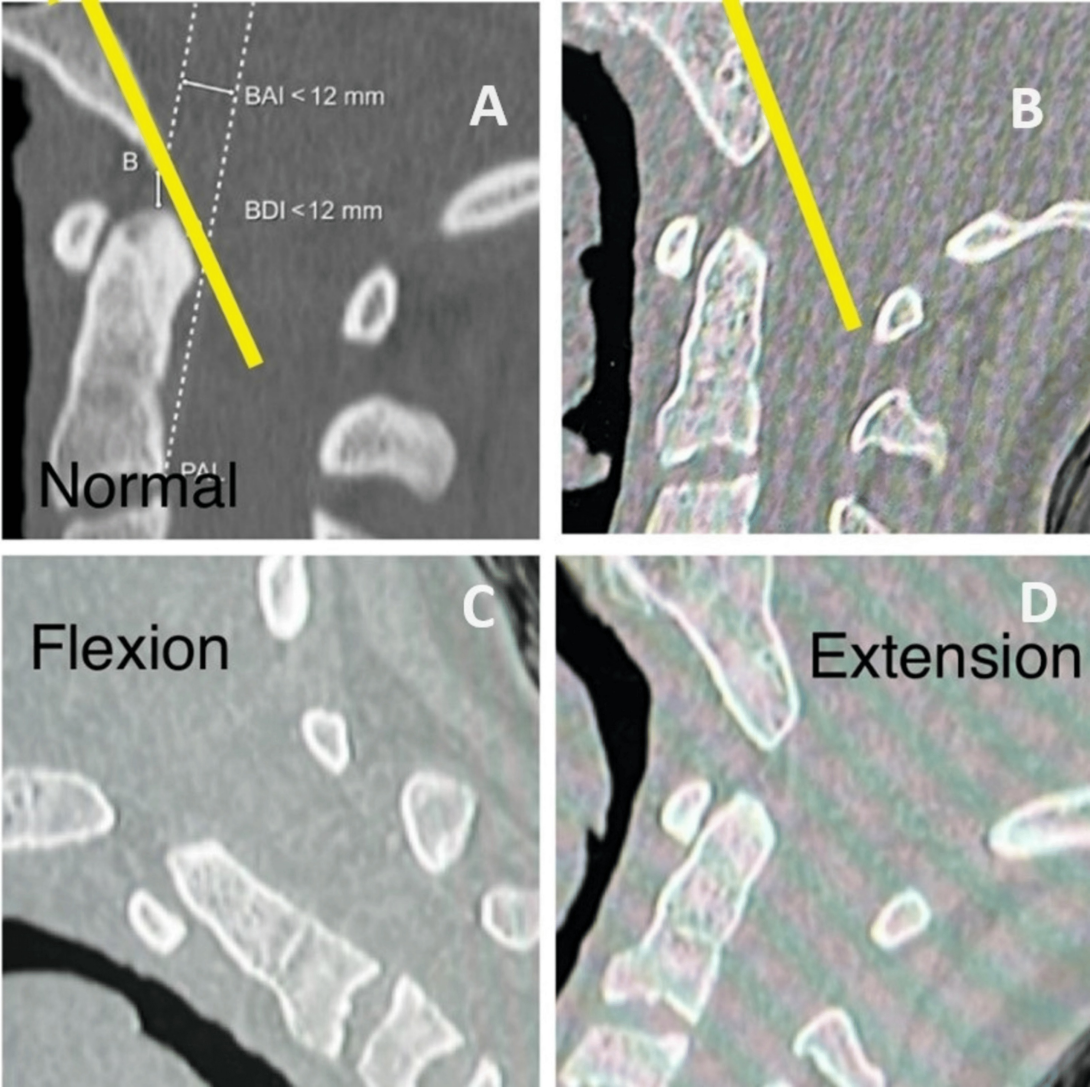
The patient’s CT scan showed a widening of the basion-dens interval (BDI) with vertical dissociation in neutral (B), flexion (C), and extension (D) views compared to normal one (A).

Plain radiographs including anteroposterior view, lateral flexion, and lateral extension views of the cervical spine revealed instability in the patient’s craniocervical region with subluxation and instability noted at the atlantooccipital (C0/C1) joint (Figures 1, 2). The patient was urgently taken for computed tomography (CT) and magnetic resonance imaging (MRI), where a series of axial cuts through the craniocervical region were obtained (Figure 3). The report showed a widening of the basion-dens interval measuring 12mm with vertical dissociation, suggestive of type 2 craniocervical dissociation (Figure 4). Additionally, a linear high signal intensity was observed in the left alar ligament indicative of a tear. Disruption of the apical and cruciate ligaments was noted as well.

Without delay, the patient was immediately planned for emergency surgery. The surgery was performed under general anesthesia with continuous monitoring utilizing neuro-electrophysiological monitoring. The patient was carefully transferred to the operating theatre with a cervical collar in place to protect her neck. Upon arrival, she was gently positioned in a prone position on the operating table and her skull was stabilized using the Mayfield skull clamp. The cervical collar was then removed before the surgery.

**Figure 5:**
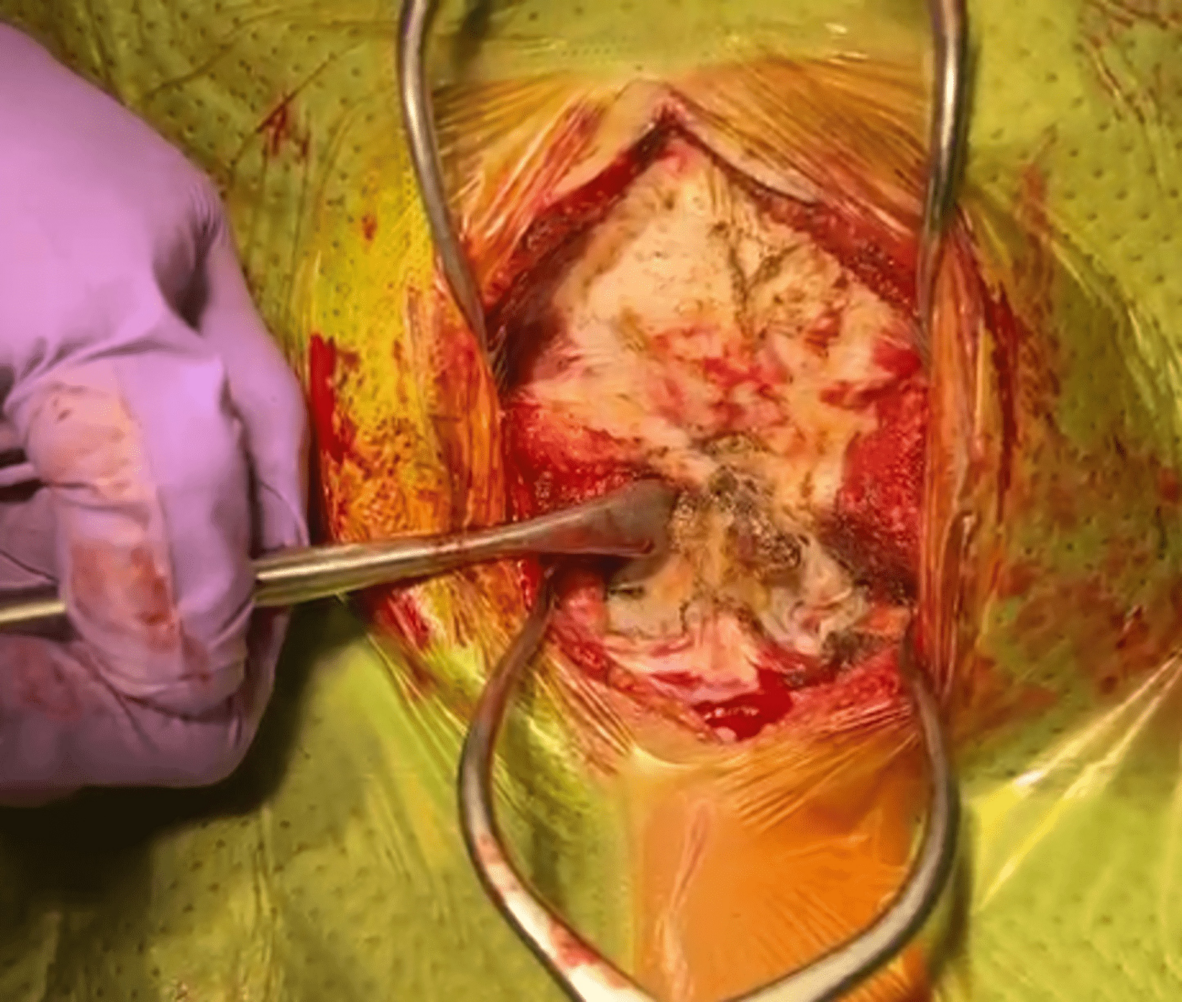
Post-reduction C2 aligned to external occipital protuberance.

The level of the vertebra was identified under image intensifier guidance. A midline incision was made over the posterior upper cervical region and the surgical wound was opened in layers to expose the spinous process of C1 up to the C3 cervical spine. The muscle attachments were dissected to expose the posterior arch and lateral processes of the cervical vertebrae. Intraoperatively, it was found that there was atlantooccipital (C0/C1) subluxation and instability along with laxity of the facet joints. However, the dural sheath remained intact (Figure 5). 

**Figure 6:**
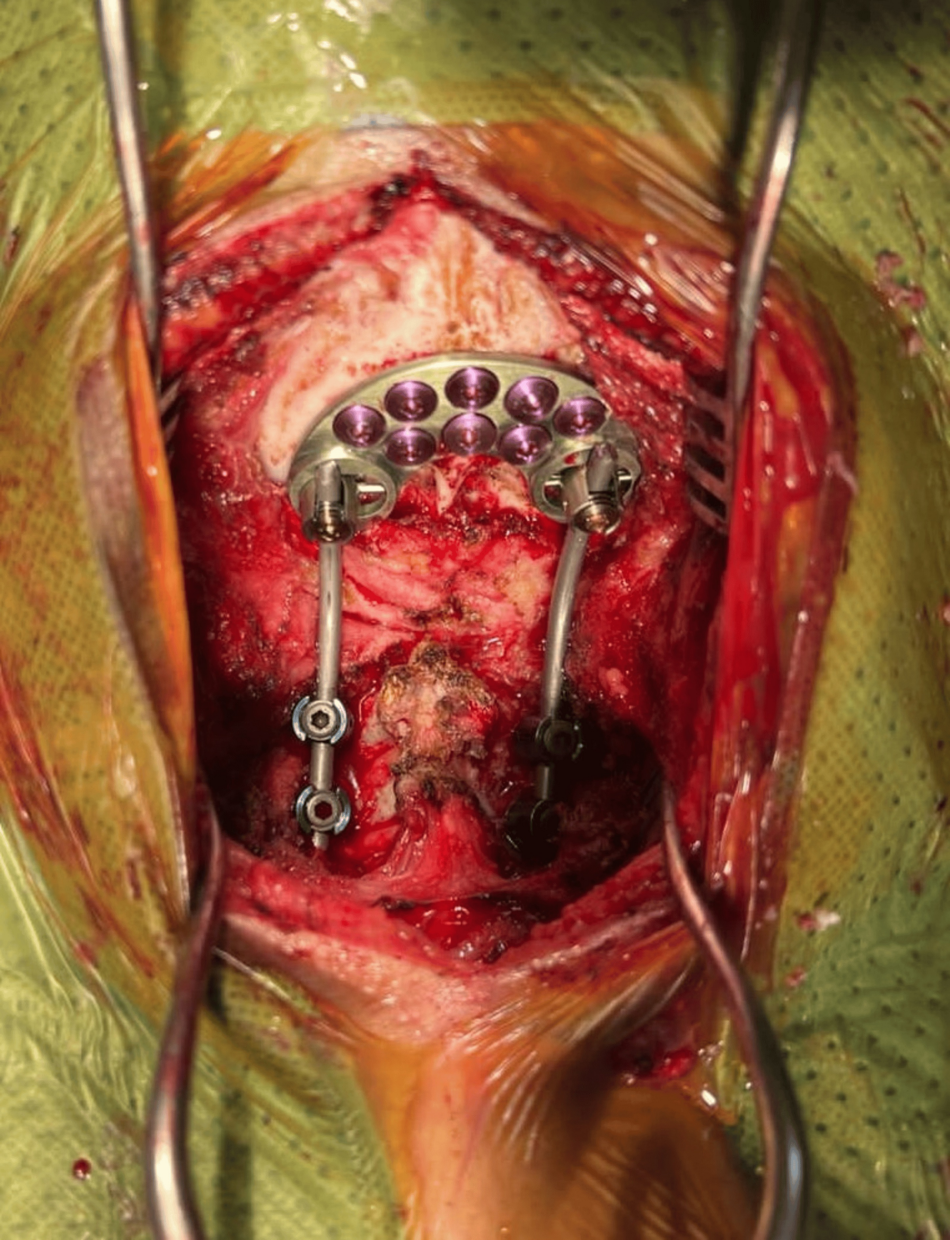
Posterior fusion occiput to C2 and C3.

An occipital plate was applied over the occipital bone and secured with screws, followed by the insertion of lateral mass screws over the C2 and C3 cervical spine. The coronal subluxation of both the atlantooccipital (C0/C1) cervical joint was then reduced and aligned using rods. Posterior fusion of the occiput to C2 and C3 was done (Figure 6). Subsequently, the placements of screws and rods post-reduction and stabilization were checked under image intensifier (I/I) guidance. A demineralized bone matrix (DBM) was inserted before the closure of the surgical wound. Skin closure was completed using the subarticular suturing method with Monosyn 2/0 after the surgery.

Two spine surgeons simultaneously performed the exposure of the cervical spine, insertion of the occipital plate with screws, and lateral mass screw insertion. The total operating time was one hour and 30 minutes and the patient remained hemodynamically stable throughout the procedure.

**Figure 7:**
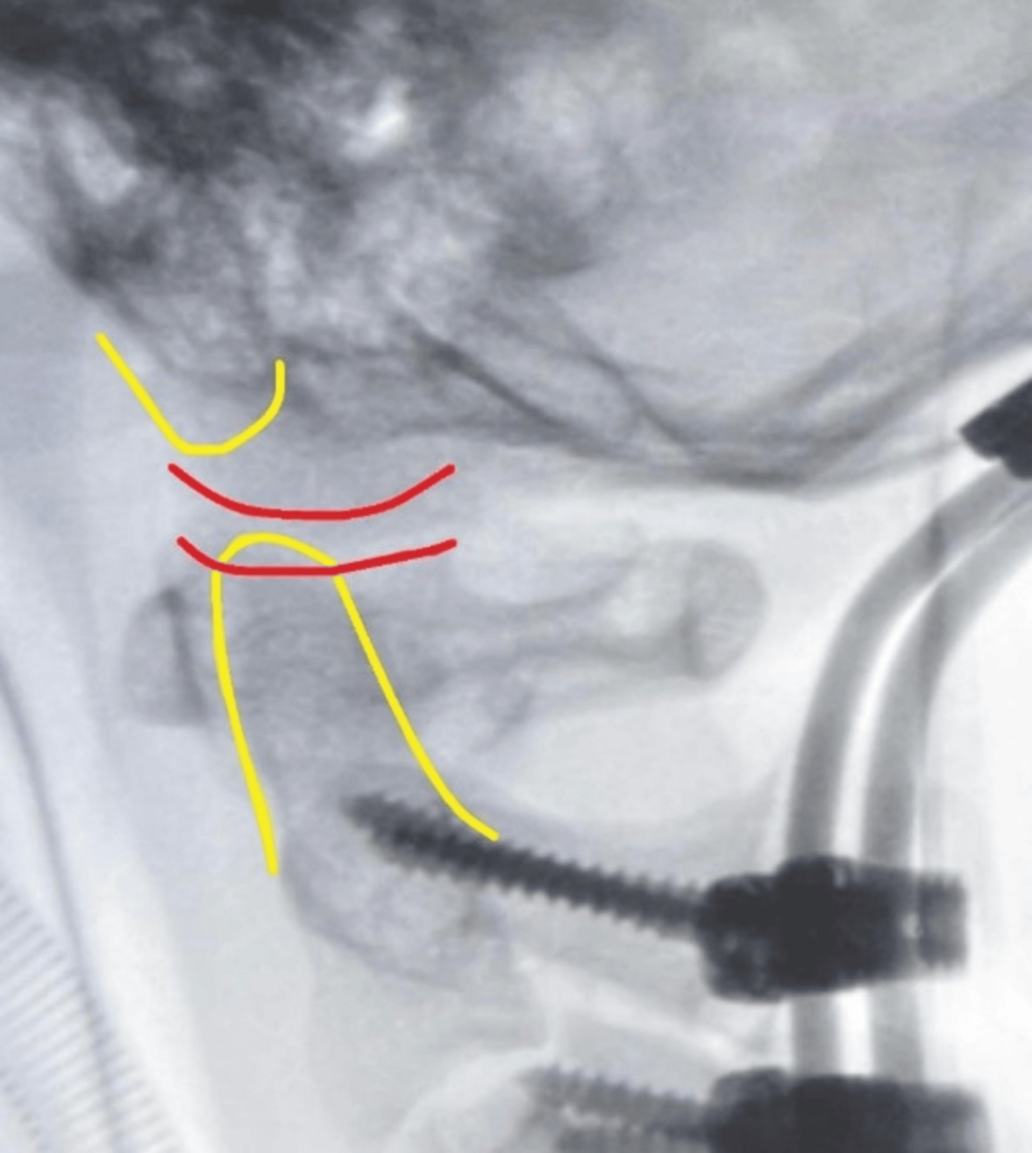
Cranio-cervical alignment restored post-fusion surgery.

Post-operative cervical spine radiographs taken under an image intensifier revealed satisfactory screw placement, acceptable spinal alignment, and reduced subluxation of the atlantooccipital (C0/C1) cervical joints (Figure 7). The patient was then transferred back to the ward postoperatively for monitoring.

Postoperatively, the patient showed stable vital signs and received pain control treatment under the co-management of the acute pain service team. The subluxation of the atlantooccipital (C0/C1) cervical joint was completely reduced. In the present case, we achieved satisfactory correction of the cervical spine alignment and adequate reduction of the subluxated craniocervical spine joints in a single-stage surgery during the acute presentation of the patient, resulting in a favorable outcome and neurological recovery for the patient. Upon discharge from the spine ward, the patient was referred to physiotherapy and spine rehabilitation units for further care and rehabilitation.

**Figure 8:**
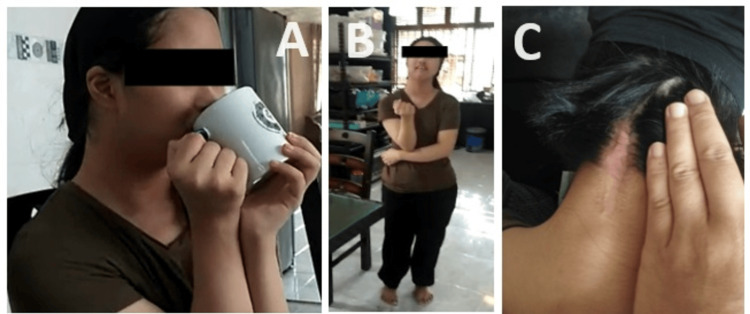
The patient demonstrated good neurological recovery and healed surgical scar during the one-year follow-up. The patient was able to drink by herself (A) and ambulate well without aid (B). No more floppy neck seen. The surgical scar was completely healed (C).

**Figure 9:**
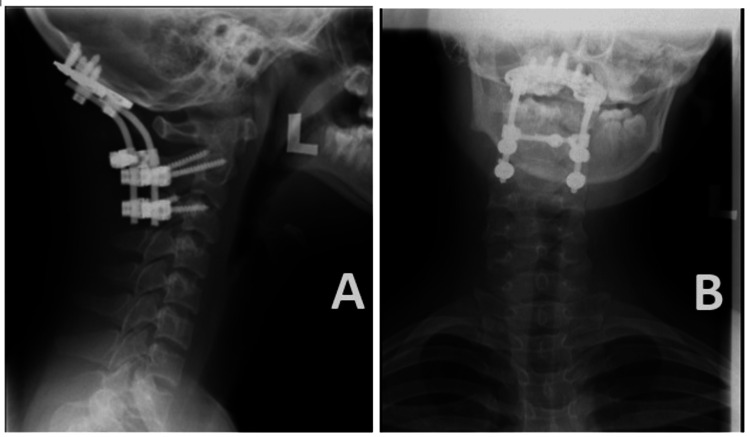
One-year follow-up cervical radiograph revealed that implants were in situ with no signs of loosening and the cranio-cervical alignment was well maintained. Lateral view (A) and anteroposterior view (B) of the cervical radiograph.

At the one-year follow-up in our spine clinic, the patient demonstrated excellent neurological recovery, with notable improvements in urinary and bowel symptoms. The patient no longer exhibited a floppy neck and she displayed good motor power in all limbs. The wound over the posterior neck completely healed. Additionally, she regained the ability to feed herself by holding cutlery and utensils independently. The patient demonstrated the capability to ambulate independently without assistance at home (Figure 8). Follow-up x-rays revealed that all implants were in situ and maintained good craniocervical alignment. The cranio-cervical joint fusion remained intact without any signs of subluxation or implant loosening (Figure 9).

## Discussion

The abnormality of atlantooccipital instability in patients with Down syndrome is often attributed to ligamentous laxity. The atlantooccipital (AO) joint is inherently shallow and prone to instability. Typically, motion at the joint is restricted to approximately 13 degrees of flexion-extension and eight degrees of lateral bending, with minimal rotation or translation [5]. Several ligaments play a crucial role in stabilizing the AO joints, with the most significant being the intraspinal ligaments [6]. These include the tectorial membrane, the suspensory ligament of the atlas, and the two alar ligaments. The tectorial membrane is the cephalad continuation of the posterior longitudinal ligament that extends over the odontoid to the clivus. The suspensory ligament of the atlas is extremely strong and extends from the tip of the odontoid to the midpoint of the anterior margin of the foramen magnum. The alar ligaments extend from the upper and lateral portions of the odontoid to the medial aspects of the occipital condyles. The extraspinal ligaments which include the anterior and posterior AO ligaments are less important than the intraspinal ligaments. They, along with the tectorial membrane, limit flexion and extension of the AO joints, while the alar ligaments are primarily responsible for limiting lateral flexion [7].

Tishler first proposed that laxity of the transverse ligament possibly as part of a generalized increase in ligamentous laxity observed in Down syndrome could contribute to this condition. Additionally, abnormal development of the odontoid process has been implicated in predisposing individuals to cranio-cervical subluxation, with an increased incidence of such abnormalities noted in Down syndrome. These abnormalities include odontoid dysplasia, hypoplastic odontoid, os odontoideum, ossiculum terminal, and a "third condyle" or manifestation of occipital vertebrae [8].

The basic cervical x-ray, including anteroposterior, lateral, flexion, and extension views, is crucial for suspected cases of craniocervical dislocation. Furthermore, CT scanning and MRI are useful in identifying other anomalies such as a basilar impression, an OS odontoideum, and a dysplasia of atlantooccipital and atlantoaxial joints. They can also clearly demonstrate the basion-dens interval and craniocervical dissociation as can be seen in our patient. Many different methods have been described in the literature to diagnose atlantooccipital dissociation and assess the instability of the craniovertebral junction. These include the Traynelis et al. classification [9], Powers' ratio [3], basion-dens interval, basion-axis interval, occipital condyle-C1 interval, and others [10].

The odontoid dens are normally aligned directly beneath the basion (the midsagittal point of the anterior lip of the foramen magnum) in a lateral view of the cervical spine in a neutral position. It maintains an average distance of approximately 5 mm and the distance may be extended up to 10mm in infants and children due to incomplete bone growth. The Traynelis classification for atlantooccipital dislocations is based on the position of the occipital condyles relative to the atlas: type I signifies anterior displacement, type II involves longitudinal distraction, and type III denotes posterior displacement [9]. However, this classification does not address rotational or coronal malalignment, which represents a notable limitation. Our case corresponds to Traynelis type II, characterized by vertical dissociation of the atlantooccipital joint.

Wackenheim Clivus Canal Line (WCCL), also referred to as the Basilar Line, involves drawing a line along the clivus and extending it inferiorly to the upper cervical canal. This reference line should be in contact only with the posterior aspect of the odontoid tip. The flexion or extension of the neck should not cause any changes to the line. The odontoid process transects the basilar line in the case with basilar invagination. The line will extend posterior to the odontoid process in posterior atlantooccipital dislocation and the line will extend through the center of the odontoid process or more anteriorly in anterior atlantooccipital dislocation [11]. Cervical radiograph in extension and neutral views in our patient showed subluxation of the atlantooccipital joint. Powers et al. calculated a ratio by measuring the distances from the basion to the posterior arch of the atlas, and from the opisthion to the anterior arch of the atlas [3]. We have observed that the methods outlined by Traynelis et al. [9], Wackenheim [11], and Wholey et al. [12] are the simplest and clinically reliable. It is not always possible to identify the exact position of the basion or opisthion as shown in our case. Additionally, we also analyze the relationship between the cranial surface of the clivus and the odontoid.

Hungerford et al. (1981), reported a case of atlantooccipital & C1/C2 dislocation in Down syndrome with severe cord compression where the patient presented with quadriparesis for four months. The patient underwent posterior fusion and subsequently neurology improved [4]. Braakhekke et al. (1985), reported another case with atlantooccipital dislocation and cervical anomalies in Down syndrome. The patient presented with bilateral lower limb weakness for six months before admission. A three-week period of 5 kg halo-traction failed to induce any reduction of the dislocations or to improve the clinical signs in the patient. Subsequently, the patient underwent posterior spondylodesis from occiput to C2, using a hooked small fragment T-plate screwed to the occiput and fixed to C1 and C2 with bilateral wiring techniques and iliac bone grafts. The occipitocervical angle was normalized following the surgery. A light halo-traction was continued for a few weeks postoperatively and the clinical picture of the patient was improving [13].

According to Rosenbaum et al. (1986), two patients with underlying Down syndrome were reported with atlantooccipital subluxation. The first patient was diagnosed upon cervical screening for mild neck pain. The second patient was incidentally diagnosed during the cervical spine evaluation prior to beginning training for the Special Olympics. However, the patient was asymptomatic and active. The first patient defaulted on the follow-up but returned eight months later due to increasing neck pain and discomfort. Operative fusion surgery of the occiput and posterior cervical spine was planned for the patient; however, the report lacks further details regarding the specifics of the surgery and the patient's recovery symptoms. The second patient was treated conservatively with three monthly monitoring and follow-up. The patient remained asymptomatic [14].

In 1991, Stein et al. reviewed and summarized the clinical and radiographic findings of fourteen cases of AOS in Down syndrome: five patients were seen at their institution and the other nine patients were described in previous literature. The majority of these patients were asymptomatic and were diagnosed through routine screening. Only two patients presented with neurological symptoms (difficulty in walking), and one patient experienced occasional neck pain [7]. The only two patients with neurological deficits presentation among the fourteen patients underwent posterior occipital-cervical fusion surgery as reported by Hungerford [4] and Braakhekke [13] while the rest were treated conservatively through observation and close follow-up.

Treatment of AOS should commence at the scene or in the emergency department as soon as the diagnosis is clinically suspected. Cervical immobilization should be promptly initiated as the first measure while awaiting further radiographic investigation. In some cases, preoperative reduction of dislocation can be achieved through traction. Posterior fixation remains the preferred treatment choice in symptomatic patients or craniocervical instability as shown in our case. There is no direct correlation between dislocation reduction and surgical outcomes. It appears justified to proceed with posterior fixation even if complete reduction is not achieved. In this case, posterior decompression should be avoided as it may lead to cervical instability while providing only minimal resolution for the anterior compression on the spinal cord. The clinical picture of our patient greatly improved postoperatively.

As healthcare providers, we must pay closer attention to craniovertebral malformations in patients with Down syndrome. The majority of patients with Down syndrome and AOS exhibit no symptoms or neurological findings. Symptomatic patients are usually observed in those with atlantoaxial subluxation (AAS) or other multiple cervical anomalies. Although AOS is less common than AAS in children with Down syndrome, a better understanding of its clinical significance should be attained. There is currently no definitive method established as the gold standard for diagnosing AOS, leaving clinicians susceptible to misdiagnosing AOS due to its subtle clinical manifestations and no radiographic measures can entirely rule out the diagnosis. The majority of patients with Down syndrome who have AOS or AAS are both asymptomatic and free of neurological findings. Early detection, diagnosis making, and surgical planning will improve surgical outcomes and quality of life for these groups of patients.

## Conclusions

In conclusion, non-traumatic AOS in patients with Down syndrome is exceptionally rare, often leading to its diagnosis being overlooked or delayed. Patients with subluxation and displacement of craniocervical joints are usually asymptomatic in the early stages and can occur without any traumatic causes reported in history taking. Orthopedic surgeons should maintain a vigilant stance regarding the potential for atlantooccipital joint displacement in patients with Down syndrome. Hence, thorough physical examination and extensive radiological investigation of the craniocervical region are necessary to prevent delayed or missed diagnosis.

An early diagnosis with appropriate pre-operative planning is the key to success in reducing and stabilizing the cranio-cervical displacement in patients with underlying Down’s syndrome. This approach can lead to improvement in neurological deficits and achieve excellent long-term surgical outcomes. The posterior occipital-cervical fusion surgery is a successful surgical strategy for managing patients with unstable AOS in patients with underlying Down syndrome, especially those who present with neurological deficits. Surgical intervention in this case not only enhances the patient’s quality of life but is also essential.
